# A plastic and reconstructive surgery landscape assessment of Malawi: a scoping review of Malawian literature

**DOI:** 10.1186/s40001-022-00714-y

**Published:** 2022-07-12

**Authors:** Chifundo Msokera, Meredith Xepoleas, Zachary J. Collier, Priyanka Naidu, William Magee

**Affiliations:** 1 Operation Smile Inc, Virginia Beach, Virginia USA; 2 University of Malawi College of Medicine, Blantyre, Malawi; 3 Division of Plastic and Reconstructive Surgery, Keck School of Medicine of USC, Los Angeles, CA USA; 4 Division of Plastic and Maxillofacial Surgery, Children’s Hospital Los Angeles, Los Angeles, CA USA; 5 Department of Plastic Surgery, Shriners Hospital for Children, Los Angeles, CA USA; 6 Operation Smile Malawi, Area 6, P.O BOX 484, Lilongwe, Malawi

**Keywords:** Malawi, Plastic surgery, Reconstructive surgical procedures, Burns

## Abstract

**Background:**

Plastic and reconstructive surgery (PRS) remains highly relevant to the unmet need for surgery in Malawi. Better understanding the current PRS landscape and its barriers may help address some of these challenges. This scoping review aimed to describe: (1) the scope and focus of the PRS literature being produced in Malawi and (2) the challenges, deficits, and barriers to providing accessible, high-quality PRS in Malawi.

**Methods:**

This scoping review was conducted on four databases (SCOPUS, PubMed, Web of Science, EMBASE) from inception through September 1, 2020 following the PRISMA-ScR guidelines.

**Results:**

The database search retrieved 3852 articles, of which 31 were included that examined the burden of PRS-related conditions in Malawi. Of these 31 articles, 25 primarily discussed burn-related care. Burns injuries have a high mortality rate; between 27 and 75% in the studies. The literature revealed that there are only two burn units nationally with one PRS specialist in each unit, compounded by a lack of interest in PRS specialization by Malawian medical students. Congenital anomalies were the only other PRS-related condition examined and reported in the literature, accounting for 23% of all pediatric surgeries in tertiary facilities.

**Conclusions:**

There is a need to increase the country's capacity to handle burn reconstruction and other PRS-related conditions to reduce overall morbidity and mortality. Additional publicly funded research at the district and community level is warranted to determine the true burden of PRS disease in Malawi to derive health system strengthening and workforce capacity building strategies.

**Supplementary Information:**

The online version contains supplementary material available at 10.1186/s40001-022-00714-y.

## Background

Approximately five billion people worldwide lack access to safe, timely, and affordable emergency and essential surgical care [1]. Most of the capacity deficit is in low- and-middle-income countries (LMICs), where the poorest one-third of the world’s population receives only 3.5% of all surgical procedures [2]. The lack of surgical care takes a serious human and economic toll and can lead to acute, life-threatening complications as well as severe psychological sequelae [3]. In other instances, poor quality care results in life long disabilities and morbidities that make productive employment less likely and imposes a financial burden on family members and society [4]. The global disparity in access to surgical care is often due to a lack of prioritization of surgical services in LMICs to address this important public health problem [4]. Thus, surgically treatable conditions such as obstructed labor [5, 6], traumatic injuries [6, 7], intra-abdominal emergencies [8], and congenital conditions (e.g., clubfoot and cleft lip or palate) [9, 10] remain commonly untreated in LMICs [11].

The lack of data on the burden of surgical diseases and injuries in Malawi compromises effective health sector planning and perpetuates the current underfunding of surgical services [12]. Surgical conditions have received little attention from public health planners in LMICs, such as Malawi [13]. The burden of surgical pathologies and access to surgical care has not been adequately identified in many LMICs. Therefore, additional information on the spectrum and burden of surgical disease in Malawi is important to uncover the unmet need for surgery and for planning of the National Health Service [12]. Comparable to other sub-Saharan African countries, 35% of the population in Malawi is living with a condition needing surgical consultation or intervention, and 24% of all deaths in 2016 were potentially avoidable with surgery [12]. Major organizations, such as the World Health Organization, have lobbied for the prioritization of surgery in public health planning to urgently scale up surgical services and training to address surgically treatable conditions within the country [14].

Plastic surgery remains highly relevant to this unmet need for essential surgery in Malawi. Some of the most common injuries in Malawi include road traffic accidents and burns, which often require Plastic and Reconstructive Surgery (PRS) for effective and comprehensive treatment [15]. In addition, in 2017, it was reported that 11% of all newborns in Malawi have congenital malformations [16]. There are many challenges to providing PRS in resource-constrained environments, such as a lack of PRS-trained medical personnel, inadequate surgical facilities, insufficient supporting ancillary services, ineffective referral systems, and equipment shortages [17]. To inform future research, a synthesis of the current literature was done to develop an understanding of the PRS landscape in Malawi. To date, there is no systematic or scoping review describing the PRS landscape with respect to relevant conditions, providers, and barriers to care in Malawi. This scoping review aimed to describe the scope and focus of the PRS literature produced in Malawi and the cited challenges, deficits, and barriers to care that exist for increasing access to high-quality PRS in Malawi.

## Methods

This scoping review was conducted following the Preferred Reporting Items for Systematic Reviews and Meta Analyses extension for Scoping Reviews (PRISMA-ScR) guidelines for reporting [18]. A detailed protocol was published on Open Science Framework on August 25, 2020 [19].

### Research questions

Through the available literature, this review attempted to answer the following two questions: (1) what is the scope and focus of existing PRS Malawian literature? and (2) what are the challenges cited in the literature to providing PRS in Malawi? PRS Malawian literature was defined as any published article examining a factor of PRS-related healthcare in Malawi with at least one author affiliated with a Malawian hospital or institution.

### Research criteria

Included articles were original studies published in English that addressed PRS providers, pathologies, or care in Malawi with at least one author affiliated with a Malawian hospital or institution. PRS providers were defined as PRS or Oral Maxillofacial surgeons, residents, clinical officers, and students pursuing the PRS or Oral Maxillofacial fields. All studies examining diseases typically treated by PRS such as burns, oncologic reconstruction, congenital anomalies, and trauma were included. All original study designs were included, but grey literature (editorials, personal anecdotes, theses, dissertations, and books) was excluded. No restriction was placed on the year of publication.

### Search strategy, study selection, and data collection

A search of SCOPUS, PubMed, Web of Science, and EMBASE was conducted on September 1, 2020, using sixteen constructs (Additional file 1). The results were downloaded into Microsoft Excel 2010 (Microsoft Corporation) and duplicates were removed. One author (M.X.) conducted the initial review and excluded articles that did not meet the inclusion criteria according to title only. The remaining articles were independently reviewed against the inclusion criteria by two authors (C.M., M.X. P.N. or Z.C.) according to their abstracts. Differences between authors were resolved through discussion. The remaining articles were input into a chart created on REDCap (Version 10.3.5, Vanderbilt University, 2004). This REDCap chart was a series of questions and short answers that was used to extract the relevant study characteristics: study design, study setting, publication year, study population, PRS category, and the study’s main findings (see Additional file 2). The full text articles were reviewed by two authors (C.M., M.X. P.N. or Z.C.). Each author input study characteristics into the REDCap questionnaire as they reviewed the full text to confirm the study’s relevance to the research question. Studies that did not meet the inclusion criteria were excluded and reasons for exclusion were recorded in REDCap. A final list of included studies was compiled by resolving any differences between the authors’ final lists through discussion. Data from the included studies were downloaded from REDCap and compiled in Excel for analysis.

### Synthesis of results

**Fig. 1:**
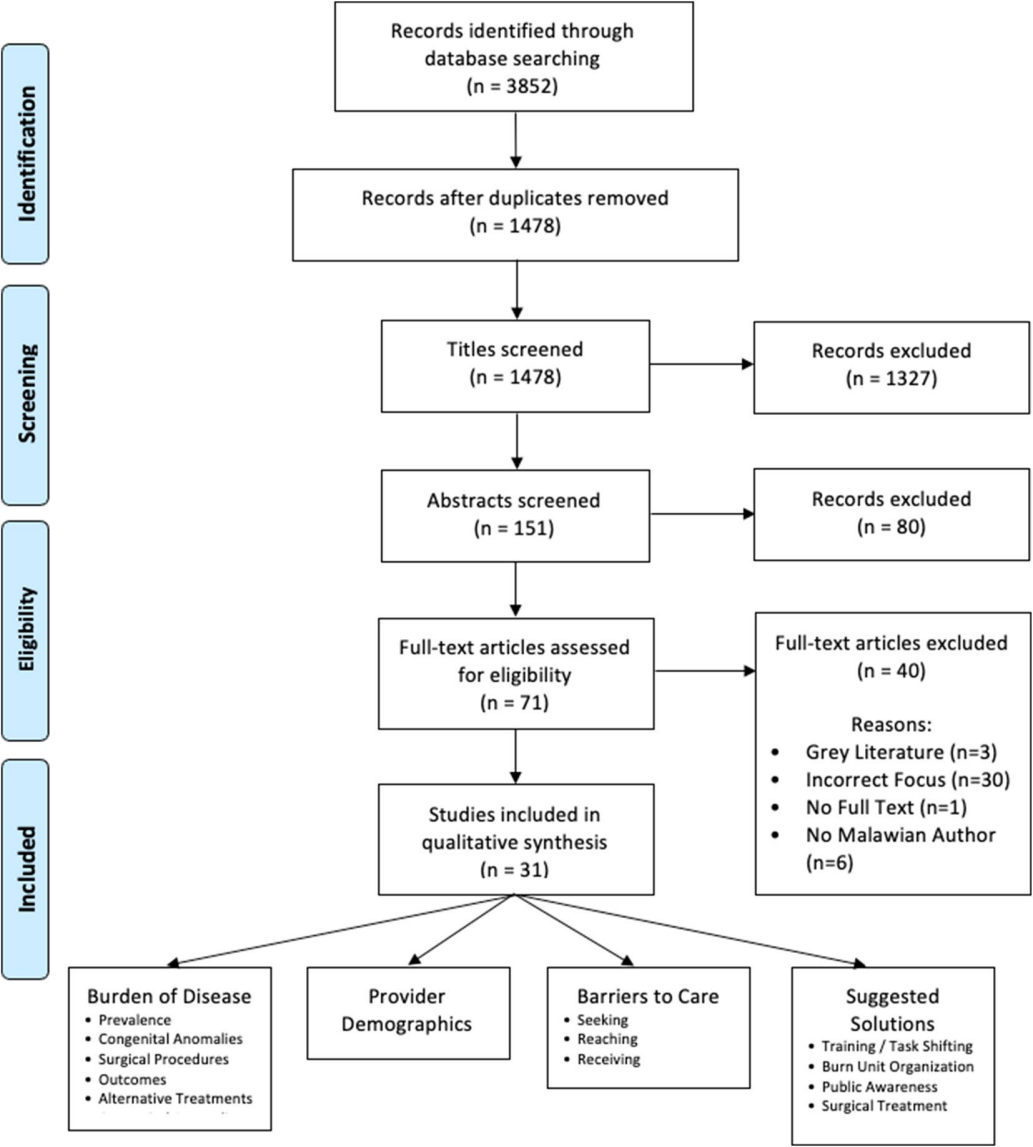
PRISMA flowchart of review stages

Study data were sorted into four main thematic categories: epidemiology, provider demographics, barriers to care, and suggested solutions. Studies could contain data belonging to multiple categories (Fig. 1). During analysis, themes were discovered within each major category (Fig. 1). Summative and descriptive statistics were used to describe the findings retrieved from the studies included in the review.

## Current status of knowledge

**Fig. 2:**
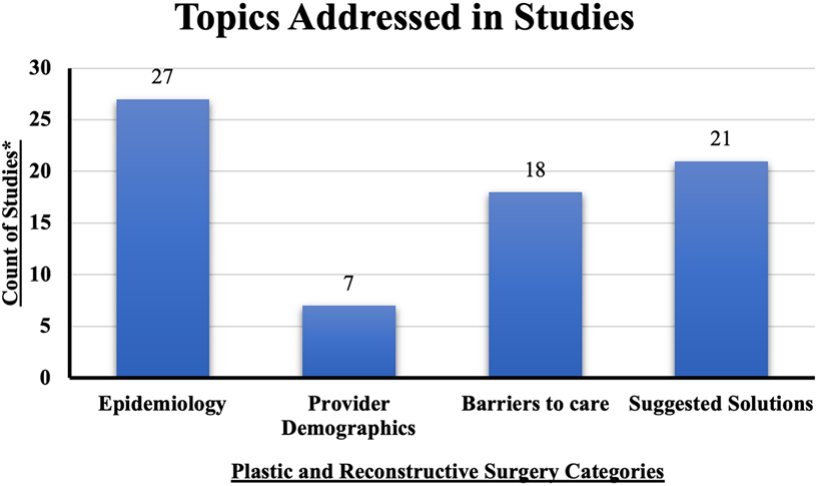
Categories of the 31 studies focusing on PRS in published Malawian literature. *Studies could be assigned to multiple categories

The database search retrieved a total of 3852 articles. After full review, 3821 articles were excluded due to the following: duplicates, grey literature, incorrect study focus, lack of full text availability, or not having an author affiliated with an institution in Malawi. This process resulted in 31 studies that met the inclusion criteria and were published between 2003 and 2020 (Fig. 1 and Table 1). The demographics of the studies are included in Table 2 and Fig. 2.

**Table 1:** Studies included in scoping review

Title	Year	Plastic and reconstructive category(s)	Author(s) affiliated with a Malawian Institute	Study location
Bacteriology of burns at the Queen Elizabeth Central Hospital, Blantyre, Malawi [47]	2003	Barriers to care	First author	Queen Elizabeth Central Hospital
		Suggested solutions	Last author	
Epidemiology and mortality of burns at the Queen Elizabeth Central Hospital Blantyre, Malawi [32]	2003	Epidemiology	First author	Queen Elizabeth Central Hospital
		Suggested solutions	Last author	
The prevalence of HIV infection among burn patients in a burns unit in Malawi and its influence on outcome [34]	2003	Epidemiology	First author	Queen Elizabeth Central Hospital
		Barriers to care	Last author	
Epidemiology of paediatric trauma admissions at Queen Elizabeth Central Hospital, Blantyre [28]	2005	Epidemiology	First author	Queen Elizabeth Central Hospital
			Last author	
Presentation and management of burn injuries in Malawi [29]	2006	Epidemiology	Last author	Queen Elizabeth Central Hospital
		Provider demographics		
		Barriers to care		
		Suggested solutions		
Burns in Malawi [30]	2006	Epidemiology	First author	Queen Elizabeth Central Hospital
		Provider Demographics	Last author	
		Suggested solutions		
A national survey of surgical activity in hospitals in Malawi [41]	2006	Epidemiology	Middle author	–
Epidemiology and bacterial colonization of burn injuries in Blantyre [31]	2007	Epidemiology	First author	–
		Barriers to care	Last author	
		Suggested solutions		
Factors involved in selection of a career in surgery and orthopedics for medical students in Malawi[46]	2010	Provider demographics	Last author	–
The epidemiology, management, outcomes and areas for improvement of burn care in central Malawi: an observational study [22]	2011	Epidemiology	First author	Kamuzu Central Hospital
		Barriers to care	Last author	
		Suggested solutions		
Epidemiology of pediatric injury in Malawi: burden of disease and implications for prevention [27]	2012	Epidemiology	Middle author	Kamuzu Central Hospital
Survival after burn in a sub-Saharan burn unit: challenges and opportunities [23]	2013	Epidemiology	Last author	Kamuzu Central Hospital
		Barriers to care		
		Suggested solutions		
Pediatric surgical care in Lilongwe, Malawi: outcomes and opportunities for improvement [43]	2014	Epidemiology	First author	Kamuzu Central Hospital
		Provider demographics	Last author	
		Barriers to care		
		Suggested solutions		
Delivery of operative pediatric surgical care by physicians and non-physician clinicians in Malawi [38]	2014	Epidemiology	First author	–
		Barriers to care	Last author	
		Suggested solutions		
The role of seizure disorders in burn injury and outcome in Sub-Saharan Africa [33]	2014	Epidemiology	Last author	Queen Elizabeth Central Hospital
		Suggested solutions		
The burden of trauma in four rural district hospitals in Malawi: a retrospective review of medical records [26]	2014	Epidemiology	Middle author	4 district hospitals: Dedza, Mangochi, NkHata Bay, and Thyolo
Burn care delivery in a sub-saharan african unit: a cost analysis study [21]	2015	Epidemiology	Middle author	Kamuzu Central Hospital
		Provider demographics		
		Barriers to care		
Surgical and anaesthetic capacity of hospitals in Malawi: key insights [49]	2015	Provider demographics	Last author	–
Qualitative evaluation of paediatric burn injury in Malawi: assessing opportunities for injury prevention [35]	2016	Epidemiology	Last author	Kamuzu Central Hospital
		Suggested solutions		
The effect of seasonality on burn incidence, severity and outcome in Central Malawi [24]	2017	Epidemiology	First author	Kamuzu Central Hospital
		Barriers to care	Last author	
		Suggested solutions		
The effect of pre-existing malnutrition on pediatric burn mortality in a sub-Saharan African burn unit [36]	2017	Epidemiology	First author	Kamuzu Central Hospital
		Barriers to care	Last author	
		Suggested solutions		
Untreated surgical conditions in Malawi: a randomised cross-sectional nationwide household survey [12]	2017	Epidemiology	First author	–
		Barriers to care		
		Suggested solutions		
Pre-burn malnutrition increases operative mortality in burn patients who undergo early excision and grafting in a sub-Saharan African burn unit [39]	2018	Epidemiology	First author	Kamuzu Central Hospital
		Barriers to care	Last author	
		Suggested solutions		
Colonization with Multidrug-Resistant Enterobacteriaceae is Associated with Increased Mortality Following Burn Injury in Sub-Saharan Africa [25]	2018	Epidemiology	Last author	Kamuzu Central Hospital
		Suggested solutions		
Patterns, quality and appropriateness of surgical referrals in Malawi [37]	2020	Epidemiology	Middle author	Queen Elizabeth Central Hospital
		Barriers to care-Suggested solutions		
The effect of traditional healer intervention prior to allopathic care on pediatric burn mortality in Malawi [44]	2020	Epidemiology	Last author	Kamuzu Central Hospital
		Barriers to care		
		Suggested SOLUTIONS		
First aid management of paediatric burn and scald injuries in Southern Malawi: a mixed methods study [45]	2020	Epidemiology	Middle author	Queen Elizabeth Central Hospital
		Barriers to care		
		Suggested solutions		
Access to Operative Intervention Reduces Mortality in Adult Burn Patients in a Resource-Limited Setting in Sub-Saharan Africa [20]	2020	Epidemiology	Middle author	Kamuzu Central Hospital
		Provider demographics		
		Suggested solutions		
The Effect of Surgical Intervention on Pediatric Burn Injury Survival in a Resource-Poor Setting [40]	2020	Epidemiology	Last author	Kamuzu Central Hospital
		Suggested solutions		
Epidemiological Comparisons and Risk Factors for Pre-hospital and In-Hospital Mortality Following Traumatic Injury in Malawi [42]	2020	Epidemiology	Last author	Kamuzu Central Hospital
		Barriers to care		
Secondary overtriage of trauma patients to a central hospital in Malawi [48]	2020	Barriers to care	Middle author	Kamuzu Central Hospital

**Table 2:** Demographics of included studies

Study demographics	*N* = 31 studies
Study setting, *n* (%)	
District hospital	4 (13%)
Secondary hospital	0
Tertiary hospital	25 (80.6%)
Community	1 (3.2%)
Not applicable to study	1 (3.2%)
Identified study location, *n* (%)	
Kamuzu central hospital	15 (48%)
Queen Elizabeth hospital	9 (29%)
District hospitals	1 (3.2%)
Not reported/applicable	6 (19.4%)
Authors Affiliated with a Malawian Institute, *n* (%)	
First author only	1 (4%)
Last author only	10 (31%)
Both first and last author	12 (39%)
Neither first nor last author	8 (26%)
Patients, *n* (%)	*N* = 25 studies
Pediatric and adult patients	16 (64%)
Pediatric patients only	8 (32%)
Adult patients only	1 (4%)

## Burden of diseases (epidemiology)

### Prevalence

**Fig. 3:**
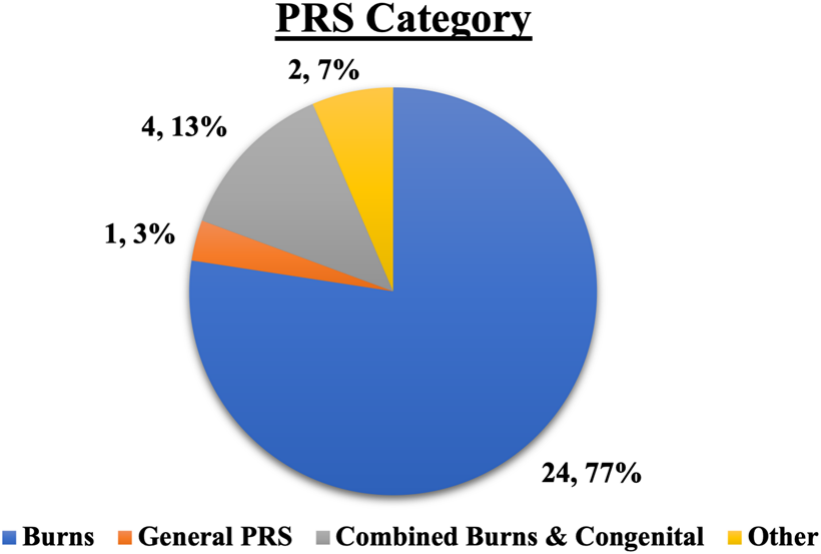
Category of PRS among published Malawian literature

The greatest burden of PRS disease in Malawian literature was primarily burn-related (Fig. 3). The prevalence of burns was reported by 17 studies [20–35]. Six of these studies reported that burns were more prevalent in younger children [21, 23, 24, 27, 32, 36]. Gallaher et al. reported that 80% of the burn patients at Kamuzu Central Hospital (KCH) were under 18-year-old during a 3-year period [21]. There was a discrepancy in the literature on the difference of prevalence of burns by sex: two studies reported more burn injuries among young males [29, 30] and one study reported more injuries among females [27]. Eight studies that reported on the types of burn found that scald burns were more common than flame burns [22, 23, 25, 28, 31–33, 35]. Another six studies, however, found that flame burns were more common among epileptic patients [27, 28, 31–34]. Burns were more common in the cold season, according to four studies that analyzed seasonality [22, 24, 26, 27].

### Congenital anomalies

Three studies examined congenital anomalies [12, 37, 38]. Pediatric patients under 15 years of age account for the majority (94%) of referrals for congenital anomalies in Malawi [37]. In a study of 2909 Malawian individuals with a mean age of 35 years, 125 (4.3%) reported having an untreated congenital anomaly [12]. Tyson et al. reported that clinical officers (CO) perform most burn surgeries, while medical doctors (MD) perform most of the cleft lip/palate surgeries [38]. Within this study the authors found that 23% of all pediatric surgical cases were performed to reconstruct congenital pathologies [38].

### Surgical procedures

Distribution and volume of surgical procedures were reported in nine studies [20–22, 25, 34, 36, 39–41]. The proportion of patients operated on varied between 19 and 30% of patients [20, 21, 25, 39, 40]. Another study by Steinlechner et al. found that skin grafting accounts for only 0.1% of the surgeries in district hospitals and only 0.6% in central hospitals in Malawi [41].

### Outcomes

Patient outcomes were reported in eleven studies [20, 22, 23, 25, 28, 31–33, 39, 42, 43]. Burn-related mortality was reported as high as 27% across all ages and as low as 11% for patients younger than 5 years [32]. The same study found that epileptic patients had a mortality rate of 16% [32]. In one study, 75% of all pediatric deaths were burn-related [28]. Kendig et al. reported that females, neonates, and patients who did not undergo surgical management were significantly more likely to die from burn-related injuries [43]. Tyson et al. found that post-operative recovery was complicated by malnutrition, HIV/AIDS, tuberculosis, malaria, inadequate access to antibiotics or analgesic for appropriate wound care [23]. In another study, more burn patients died in the hospital compared to pre-hospital setting during a 10-year period [42]. Poor nutritional status, as determined by anthropometric Z-scores, was associated with higher mortality rates, and one study found that a one-point decrease in weight-based *Z*-score correlated with a 46% increase in mortality [39].

### Alternative treatments

Two studies examined the use of alternative treatments and first aid prior to hospital care [44, 45]. One study by Gallaher et al. found that 11% of burn patients used traditional medicine prior to presenting at the hospital, and those who seek traditional methods of burn care present later to the hospital than those who do not attempt first aid [44]. Another study by Broadis et al. found that 69% of burn patients received first aid prior to hospital admittance but did not comment on timing of presentation relative to provision of first aid [45]. Type of first aid applied to burns varied widely and included the application of water, toothpaste, honey, sand, and egg on the burn injury [45].

## Provider demographics

Several studies reported only one consultant plastic surgeon in each of the country's two burn units at Kamuzu Central Hospital (KCH) and Queen Elizabeth Central Hospital (QECH) [20, 21, 29, 30]. QECH reported having one surgical trainee, while KCH reported having two COs [20, 21, 29, 30]. One study reported that there was only one fellowship-trained pediatric surgeon in Malawi [43]. In a survey of 70 Malawian medical students, only 2% indicated an interest in plastic surgery, with lack of mentorship cited as a key problem by the students [46].

## Barriers to care

### Seeking

Two studies reported on the barriers to seeking PRS, which related to inadequate knowledge [44, 45]. Gallaher et al. reported on a lack of medical literacy as a barrier to seeking and receiving burn-related care [44]. This study found that prior use of a traditional healer practitioner was associated with a delay in presentation to a burn center and an increased odds of mortality (OR 1.91, 95% CI 1.09, 3.33) [44]. Another study by Broadis et al. used qualitative interviews to demonstrate that mothers were often in charge of first aid given to burn victims and that they used intergenerational knowledge to treat the burns [45]. Most mothers used eggs as first aid for burns owing to a lack of available water in the homes and a belief that water would cause the skin to peel [45]. If the patients felt that they had received poor care at the hospital or previously had bad experiences at a hospital, they were less likely to seek hospital care [45].

### Reaching

One study identified a barrier to reaching burn and surgical care [12]. Varela et al. reported that transportation costs were higher for patients trying to reach tertiary hospitals from rural areas and unserviceable roads further compounded the issue of transportation [12].

### Receiving

Eleven studies reported on barriers to receiving burn and surgical care [22, 23, 29, 31, 34, 37, 38, 43, 47–49]. Maine et al. found that burn patients with lower total body surface area (TBSA) burns were at higher risk for being unnecessarily referred from a district hospital to a tertiary hospital due to overestimation of true TBSA [48]. Barriers reported by Tyson et al. included high pediatric patient volumes, delayed presentation and advanced pathology (often due to traveling long distances to hospitals with no stabilizing treatments), limited resources, and lack of trained pediatric surgeons [38]. Re-operation rate was higher for patients treated by COs (17%) compared to those who received care from physicians (7.1%), although some of this variability was attributable to the fact that certain burn patients required multiple operations due to the extensive nature of injury across their bodies [38]. Kendig et al. found a selection bias in which healthier patients were more likely to be selected for pediatric surgery in Malawi [43]. The author demonstrated that there is a tendency to operate on less complex patients, while conservatively managing or transferring more complex patients back to their homes for expectant care [43]. A lack of pediatric surgeons, pediatric anesthesia, post-operative critical care, and follow-up all contribute to the reluctance to perform surgery [43]. Other barriers include mislabeling of supplies [22], high prevalence of antibiotic resistant bacteria [47], repurposing of used surgical supplies [29], unnecessary referrals [37], and an inadequacy of healthcare access which led to a reliance on traditional medicine [23]. In a survey of 27 facilities (23 district hospitals and 4 tertiary hospitals), Henry et al. found that 93% were able to manage burns, but only 59% could perform skin grafts and only 62.5% could perform post-burn contracture release [49].

## Suggested solutions

### Training/task shifting

Three studies recommended increasing the number of surgeons and increasing training programs for surgeons and anesthetists in pediatric surgery, reconstructive care, and burn management [22, 23, 29, 43]. Two studies recommended task shifting [38, 43]. Tyson et al. advocated for task shifting by arguing that COs should perform more minor procedures to allow the consultant surgeons to focus on more complex cases, such as large burns and challenging reconstructions. The authors also argued that burn-trained COs could serve as the first line of treatment in district hospitals, which could alleviate the high rate of unnecessary referrals to tertiary hospitals, where capacity is overwhelmed by the influx of patients from peripheral areas within the system [38].

### Burn unit organization

Six studies advocated for better organization and management of interventions within the two tertiary level care burn units in Malawi [23, 24, 31, 37, 39, 47]. Recommendations included controlling the flow of human traffic in the burn unit to reduce infectious spread [23], enforcing stricter hand washing practices before and after handling a patient [23], streamlining referral processes and communication [37], frequent surveillance of bacterial profiles and antibiotic susceptibilities in burn wounds [31], and more intentional allocation of non-perishable supplies and money in anticipation for the increase in burn burden during the cold season [24].

### Public awareness

Ten studies suggested increasing public awareness towards burn safety and surgical care [12, 24, 29, 30, 32, 33, 35, 40, 44, 45]. For epileptic patients, Broadis et al. advocated for widespread use of anti-epileptic medications to reduce the number of burns sustained during epileptic seizures [45]. Other studies advocated for educational programs focused on parental vigilance during cooking and the safer use of fire to reduce the incidence of pediatric burns [24, 32, 35, 40]. Varela et al. recommended the visitation of a surgical team to rural areas in Malawi to provide community outreach to raise public awareness for surgery and facilitate referrals to decrease the backlog of untreated surgical conditions [12].

### Surgical treatment

Four studies advocated for earlier treatment of burns and the use of surgical interventions rather than non-operative management alone [20, 25, 30, 40]. These studies suggested that early excision of burn wounds significantly reduced bacterial colonization and subsequent infection [25], surgical interventions for burn wounds significantly increased the odds of survival [40], and that patients with deeper burns should be prioritized for surgery to prevent the mortality that often arises in resource-limited settings due to delayed treatment of severe injuries [20].

### Funding

**Fig. 4:**
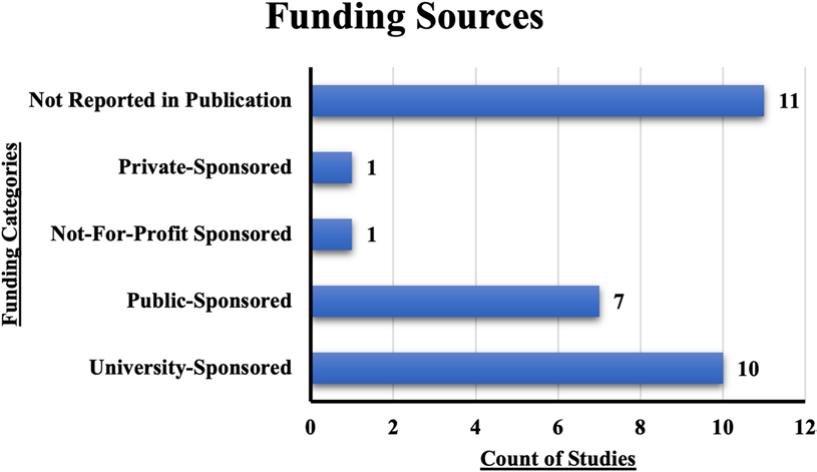
Funding sources among PRS Malawian literature

Over a third (38%) of the included studies did not report their funding source (Fig. 4). The largest proportion (32%) of studies with reported funding sources indicated funding from a university. Privately funded studies and not-for-profit organizations funded only one study each.

## Conclusions

This is the first scoping review that analyzed PRS Malawian literature. This study identified a significant shortage of literature on PRS capacity, pathologies, treatment strategies, and barriers to care in Malawi [50]. Only 31 published articles examining a factor of PRS-related healthcare in Malawi with at least one author affiliated with a Malawian hospital or institution, were identified.

Our review found that most of the literature focuses on burns with lower publication rates for trauma and congenital conditions. Because burns account for much of the PRS disease burden in Malawi, the study of burn-related injuries is pertinent to identifying challenges with burn prevention, treatment, and reconstruction that can be addressed to reduce morbidity and mortality within the country [20–35]. The high incidence of burn-related injuries is due to multifactorial processes related to sociodemographic factors, housing regulations, preexisting conditions, patient age, and health system infrastructure and capacity [51–54]. The use of open flames for cooking and heating, high density housing situations often with multi-generational occupancy, and lack of residential and industrial construction regulations are all risk factors that contribute to the high incidence of burn injury within Malawi, especially in pediatric and epileptic patients. In addition to preventing burns, providing safe alternatives to open fire cooking may help address other reconstructive issues as one recent study found that pregnant women exposed to cook smoke from open fire had higher risk of birthing children with congenital anomalies [55]. Suggested solutions in the literature include the use of public awareness campaigns for parents to encourage altered behaviors and instill safe practices around open fire cooking and heating to reduce the incidence of pediatric burns. An additional solution could be to introduce safer cookstoves that avoid open flames to reduce the risk of flame injury and congenital anomalies, which account for 23% of all pediatric surgeries in tertiary facilities [38]. Other solutions include community outreach programs supported by the Ministry of Health to broadly distribute safe cooking and heating practices, alternative heating methods that utilize protected/covered flames, and safer cooking utensils (i.e., pots with locked tops) to prevent accidental spills and scalds.

This review found that there are only two burn units in the entire country, which have severe resource constraints and multifactorial barriers to delivering optimally timed surgical care. The lack of well-resourced burn units is compounded by the fact that there are roughly two PRS surgeons for a country of 18.63 million people, which equates to a provider density of 0.01 per 100,000 residents. In contrast, the United States and Peru have PRS provider densities of 2.10 and 1.92 PRS providers per 100,000 residents, respectively [56]. This lack of surgical providers creates a significant deficit in the quantity of service that is available within the healthcare system, creating an over-burdened surgical ecosystem relying on alternative methods and avenues of treatment that significantly contributes to the poor outcomes seen in the management of PRS-related diseases. A widely suggested solution in the literature to this lack of providers is to embrace task-shifting and task-sharing in a collaborative manner by training COs to care for the majority of minor, uncomplicated surgical conditions, while MDs manage specialized and major cases requiring more complex surgical interventions. However, the majority of cases are already performed by COs, and these surgical cases are shown to have higher rates of reoperation and complications versus procedures done by MDs. As a result, there is strong evidence to support the value of adding more highly trained surgeons who would provide the complex care as well as improving training for COs who are treating these PRS conditions. Yet challenges would exist even with the expansion of the surgical workforce given the findings by Kendig et al. that even among MDs there is a tendency to operate on less complicated, non-pediatric patients, while specialized and major cases are often sent home for non-operative care [43]. Additional measures to improve infrastructure, access to medical equipment, pediatric-specific training, and the referral system could allow both MDs and COs higher functioning operating rooms along with the necessary supporting surgical systems so they may have more time to dedicate to specialized, complex, and pediatric cases.

There is a dire need to increase the number of surgeons and anesthetists in pediatric surgery, reconstructive care, and burn management. However, Malawian medical students do not show a high interest in PRS, citing mentorship and lack of exposure as key reasons [46]. With the only two PRS-trained surgeons in the country already overburdened with the present patient backlog, lack of mentorship will remain a significant problem. Education initiatives to increase the number of PRS physicians could include rotations for Malawian medical students to rotate in established PRS operating rooms and hospital wards. PRS surgeons from abroad could supplement the education workforce to teach about PRS in Malawi to the students and serve as mentors. In addition, exchange programs that take medical students outside the country to increase exposure to the scope of practice in PRS could help increase medical students’ interest. A shorter term solution could utilize general surgeons and surgical trainees, who could be taught common PRS procedures during medical missions in Malawi or as part of regional academic training programs, such as the College of Surgeons of East, Central, and Southern Africa (COSECSA) [57].

Reducing the PRS burden in Malawi will also require counteracting the barriers that patients face when seeking, reaching, and receiving PRS care. Within the PRS literature, seeking-care barriers were knowledge related, which could be combatted by increasing public knowledge of burn-related and surgical care. Common reasons many Malawian patients did not seek out PRS care was that they preferred the use of traditional healers, did not recognize the severity of an injury, or had previous negative hospital experiences that deterred them from returning to a hospital. The problem could be mitigated by educating traditional healers about the basics of burn first-aid, evidence-based practices for PRS care, referral criteria for more advanced care, and the negative outcomes if late presentation occurs. Attempts to incorporate these community providers into referral pathways may increase patient comfort with the health system which would encourage patients to present to health facilities earlier for timely care.

Barriers to reaching PRS care needed to be further examined in the Malawian literature. There was only one study that reported on barriers to reaching PRS care, specifically, access to transportation and transportation costs. Increasing the ability of district hospitals to handle more level-appropriate burn-related injuries would provide patients with closer options for receiving care, thereby decreasing the transportation costs and difficulty that is present with traveling farther to central hospitals. This would also decrease the transfer rate which would decrease time to receiving care considering poorly established referral pathways contribute to delays in obtaining treatment. Moreover, the frequently cited barriers to receiving care in the literature demonstrates that even once patients reach hospitals, they often do not get appropriate care. Pediatric patients appeared to have the most barriers to receiving care as a result of the significant lack of pediatric resources and trained workforce for pediatric surgical conditions. The inability of pediatric patients to receive PRS-related care is further compounded by the fact that socioeconomic factors are causing increased rates of burn injury within this age group that overwhelms the already short-staffed pediatric system. Possible solutions include the establishment of intersectional partnerships with NGOs and universities that could facilitate sustainable support for pediatric burn care through a combination of short-term engagements providing direct patient care, longer term training efforts with local and regional exchanges and mentorship programs, and health system strengthening endeavors. There was also a high number of organization barriers that could be mitigated with better infectious disease training among staff and a better organized referral system. There is a vast gap in the literature on the management of burn cases in district hospitals. As a result, supporting further research through in-country collaborations will help better determine the true extent of PRS-related diseases and barriers to care in these settings.

Funding drives the quantity, quality, and focus of research [58]. This is mostly likely why the majority of this research was done at teaching hospitals, where support through funds and infrastructure are more readily available as opposed to in the community, where the majority of the burden of PRS conditions exists. While burns comprise a large portion of the PRS disease burden in Malawi, it is important to ensure that this focus on burns is proportionally assigned to prevent a biased concentration of resources which would limit research on other PRS diseases. There is a great need for large, community-based PRS studies on burns, congenital anomalies, oncologic reconstruction, and trauma; all of which are greatly warranted. Additional funding priority should be given to other PRS conditions along with burns.

## Limitations

This study is limited by its design as a scoping review. There was no formal evaluation of the quality of evidence or risk of bias in the studies as the goal of this analysis was to ascertain a complete assessment of the PRS Malawian literature landscape. Restricting inclusion or evaluating quality and bias of included studies was not indicated to accomplish this broader aim. Moreover, the lack of reporting on non-burn PRS conditions limits this study’s generalizability to insight on such conditions.

## Conclusions

Burn-related research comprises the vast majority of Malawian PRS literature. Studies show that widespread use of open flame sources for cooking and heating partially account for the high prevalence of pediatric and epileptic burns—cohorts that make up the majority of Malawi’s burn burden. Furthermore, women, children, and epileptics are disproportionately affected by burn injury in Malawi due to a multifactorial interplay between social, economic, political, and cultural factors. Despite the number of studies addressing burn-related epidemiology, the prevalence of other PRS-related conditions is not well described in the current body of Malawian literature. Further studies are needed to better understand this burden and identify specific barriers to delivering care for these other conditions. Creative solutions are needed to increase the number of PRS surgical providers. Collaborative approaches such as task-shifting and task-sharing with COs may be a possible short-term solution to address the gap while increasing training capacity and mentorship programs for Malawian medical students, COs, and non-PRS providers that would help expand the PRS surgery workforce. The creation of additional avenues for funding, collaboration, and research support in Malawi could increase the capacity to perform large, community-based PRS studies for burns, congenital anomalies, oncologic reconstruction, and trauma—all of which contribute significantly to the burden of morbidity and mortality within the country. The information gleaned from this scoping review has helped identify the numerous ways in which we may begin to address these health system challenges and improve the quality, safety, and accessibility of PRS care in Malawi.

What is already known about this topic:

- There is a large unmet need for Plastic and Reconstructive Surgery in Malawi, as some of the most common injuries in Malawi include road traffic accidents and burns.
- There are many challenges to providing PRS in resource-constrained environments, such as Malawi, including a lack of PRS-trained medical personnel, inadequate surgical facilities, insufficient supporting ancillary services, ineffective referral systems, and equipment shortages.

What this study adds:

- The majority of PRS literature being produced in Malawi focuses on burns and burn-care, while there is a lack of literature on other conditions, such as congenital anomalies, oncologic reconstruction, and trauma.
- The Malawian PRS literature is mostly funded by universities and focuses heavily on tertiary hospitals, further intensifying the dearth of information on PRS conditions and providers at the district and community level.
- An insufficient number of plastic and reconstructive surgeons and burn units, low medical student interest, a high prevalence of pediatric patients with no pediatric-trained plastic surgeon, high mortality and post-operative infection rates, and numerous patient barriers to care, all compound the inadequacy of Plastic and Reconstructive Surgery in Malawi.

## Supplementary Information

**Additional file 1.** Complete MeSH search strategy used on each database.

**Additional file 2.** REDCap survey to chart results.

## Data Availability

Data sharing is not applicable to this article as no data sets were generated or analyzed during the current study.
