# Supplementary material for: A plastic and reconstructive surgery landscape assessment of Malawi: a scoping review of Malawian literature
Source: Eur J Med Res. 2022 Jul 12;27:119. doi: 10.1186/s40001-022-00714-y (PMC9277806; doi:10.1186/s40001-022-00714-y)
Supplement: Supplementary file 1 — Additional file 1. Complete MeSH search strategy used on each database. [file 40001_2022_714_MOESM1_ESM.pdf]

Appendix 1. Complete MeSH Search Strategy used on Each Database.

Terms
(Surgery) AND (Malawi)
(Plastic Surgery) AND (Malawi)
(Oral Surgery) AND (Malawi)
(Reconstructive Surgery) AND (Malawi)
(Reconstructive Surgical Procedures) AND (Malawi)
(Reconstruction) AND (Malawi)
(Trauma) AND (Malawi)
(Burns) AND (Malawi)
(Skin Graft) AND (Malawi)
(Cleft Palate) AND (Malawi)
(Cleft Lip) AND (Malawi)
(Surgery Department, Hospital) AND (Malawi)
(Surgeons) AND (Malawi)
(Oral and Maxillofacial Surgeons) AND (Malawi)
(Congenital) AND (Malawi)
(Maxillofacial) AND (Malawi)
