# Supplementary material for: A plastic and reconstructive surgery landscape assessment of Malawi: a scoping review of Malawian literature
Source: Eur J Med Res. 2022 Jul 12;27:119. doi: 10.1186/s40001-022-00714-y (PMC9277806; doi:10.1186/s40001-022-00714-y)
Supplement: Supplementary file 2 — Additional file 2. REDCap survey to chart results. [file 40001_2022_714_MOESM2_ESM.pdf]

Appendix 2. REDCap Survey to Chart Results.

- First Author (Last Name et al): ____________
- Year of Publication: ____________
- Journal: ____________
- Paper Title: ____________
- Is an author affiliated with an institution in Malawi? Yes or No
    - If yes, is it the first author? Yes or No
    - Is the senior author from Malawi? Yes or No
- Reviewer 1: Include this Study? Yes or No
    - If no, reason for exclusion:
        - No Malawian author
        - Incorrect study focus
        - Grey literature
        - Other
            - If other, please list: ____________
- Reviewer 2: Include this Study? Yes or No
    - If no, reason for exclusion:
        - No Malawian author
        - Incorrect study focus
        - Grey literature
        - Other
            - If other, please list: ____________
- Study Population:
    - Surgeons
        - If yes, select PRS Surgeons, OMF Surgeons, Both, or Surgeons (undefined)
    - Surgical Residents
        - If yes, select PRS Residents, OMF Residents, Both, or Residents (undefined)
    - Clinical Officers
    - Medical Students
    - Surgical Patients
        - If yes, select Pediatric, Adult, or Both
    - Other
        - If other, please list: ____________
    - Not Applicable
- Study setting:
    - District Hospital
    - Secondary Hospital
    - Tertiary Hospital
    - Community
    - Other
        - If other, please list: ____________
    - Not Applicable
- Does this study name a Hospital(s)? Yes or No
    - If yes, list hospital(s) name: ____________
- Study Design:
    - Qualitative studies
    - Cross-sectional analyses
    - Questionnaires
    - Longitudinal analysis
    - Controlled trials
    - Case reports
    - Observational studies
    - Other
        - If other, please list: ____________
- Category of PRS:
    - Burns
    - Congenital anomaly
    - Trauma-related
    - Reconstruction
    - Other
        - If other, please list: ____________
- Does the study's finding include?
    - Epidemiology
        - If yes, describe: ____________

○ Provider Demographics
▪ If yes, describe: ____________
○ Barriers to Care
▪ If yes, describe: ____________
○ Suggested Solutions
▪ If yes, describe: ____________
• Study's Funding Source:
○ No Funding
○ Not-For-Profit Sponsored
○ University-Sponsored
○ Public-Sponsored
○ Private-Sponsored
○ Not Reported in Publication
